# Molecular Characterization and Differential Expression of an Olfactory Receptor Gene Family in the White-Backed Planthopper *Sogatella furcifera* Based on Transcriptome Analysis

**DOI:** 10.1371/journal.pone.0140605

**Published:** 2015-11-05

**Authors:** Ming He, Ya-Nan Zhang, Peng He

**Affiliations:** 1 State Key Laboratory Breeding Base of Green Pesticide and Agricultural Bioengineering, Key Laboratory of Green Pesticide and Agricultural Bioengineering, Ministry of Education, Guizhou University, Huaxi District, Guiyang, P. R. China; 2 College of Life Sciences, Huaibei Normal University, Huaibei, P. R. China; USDA-ARS, UNITED STATES

## Abstract

The white-backed planthopper, *Sogatella furcifera*, a notorious rice pest in Asia, employs host plant volatiles as cues for host location. In insects, odor detection is mediated by two types of olfactory receptors: odorant receptors (ORs) and ionotropic receptors (IRs). In this study, we identified 63 *SfurORs* and 14 *SfurIRs* in *S*. *furcifera* based on sequences obtained from the head transcriptome and bioinformatics analysis. The motif-pattern of 130 hemiptera ORs indicated an apparent differentiation in this order. Phylogenetic trees of the ORs and IRs were constructed using neighbor-joining estimates. Most of the ORs had orthologous genes, but a specific OR clade was identified in *S*. *furcifera*, which suggests that these ORs may have specific olfactory functions in this species. Our results provide a basis for further investigations of how *S*. *furcifera* coordinates its olfactory receptor genes with its plant hosts, thereby providing a foundation for novel pest management approaches based on these genes.

## Introduction

Insects can exploit chemical signals in the environment using their accurate olfactory systems, thereby mediating many important physiological behaviors, such as mate-finding, host location, and sending alarms to conspecifics. The antennae are the major olfactory organs of insects, and they possess various types of sensilla, where peripheral olfactory signal transduction events occur. At the molecular level, three main types of proteins are generally considered to be involved in odorant molecule transduction in the sensillum. First, odorants may diffuse into the sensillar lymph via pores, where odorant-binding proteins (OBPs) recognize and bind them. Second, OBPs act as transporters to transfer odorants across the sensillar lymph to reach olfactory receptors, including odorant receptors (ORs) and ionotropic receptors (IRs), which are located on the dendrites of olfactory receptor neurons (ORNs). Finally, distinct odorant-degrading enzymes act as inactivators to degrade odorants and maintain the sensitivity of ORNs [1–4].

Insect ORs were first identified in *Drosophila* using molecular biology and bioinformatics methods [5–7]. Further studies determined that insect ORs also contain seven-transmembrane domains similar to mammalian G protein-coupled receptors, but their topology is inverted with an intracellular N-terminus and extracellular C-terminus [8–10]. The OR family has undergone rapid evolution in a species-specific manner, according to studies of insect species with available genome sequences. Thus, highly variable numbers of ORs have been identified, e.g., 62 ORs in *Drosophila melanogaster* [5–7], 79 in *Anopheles gambiae* [11, 12], 170 in *Apis mellifera* [13], 259 in *Tribolium castaneum* [14], and 66 in *Danaus plexippus* [15, 16]. Only one OR is relatively conserved among species, i.e., ORco, an obligate and universal co-receptor that interacts with other ligand-specific ORs to form an ORx-ORco complex, which functions as a ligand-gated cation channels [17, 18]. Recently, many studies have focused on pheromone receptors, especially in Lepidoptera [19–23], whereas very few studies have investigated ORs in phytophagous insects.

Animal ionotropic glutamate receptors (iGluRs) are well known for their essential roles in synaptic transmission as receptors of the excitatory neurotransmitter glutamate [24]. Recently, IRs were discovered to be a new olfactory receptor family, i.e., a variant iGluR subfamily [25–27]. IRs are involved in odorant reception, as was shown by combined mis-expression experiments and through the subcellular localization of IRs in olfactory organs with chemosensory sensilla [25]. Further research showed that IRs differ from ORs in that they are are attuned to carboxylic acids and amines. In addition to *D*. *melanogaster*, expressed sequenced tag, transcriptome, and genome analyses have identified insect IRs in representative species from multiple orders, such as *Rhagoletis pomonella* (Diptera) [28], *Anopheles gambiae* (Diptera) [29], *Bombyx mori* [27], *Spodoptera littoralis* (Lepidoptera) [30], *Chilo suppressalis* (Lepidoptera) [31], and *Aphis gossypii* (Hemiptera) [32]. However, no functional data related to IRs have been reported outside *Drosophila*. Croset et al. [27] suggested that IRs can be classified into two distinct subfamilies with different ancestors: the conserved “antennal IRs” and the species-specific “divergent IRs.” The “antennal IRs” may represent the original olfactory receptor family of insects. The “divergent IRs” derived from “antennal IR” ancestors may be involved in gustation. Chemosensory IRs could have been derived from an animal iGluR ancestor.

The white-backed planthopper, *Sogatella furcifera* Horváth (Hemiptera: Delphacidae), is a notorious rice pest that migrates among many Asian countries each year, where its sap-sucking feeding style and transmission of the southern rice black-streaked dwarf virus causes severe losses in rice crops. In addition to rice crops, *S*. *furcifera* also damages many other plants, including other Gramineae, such as *Echinochloa crus-galli*, *Zea mays*, and *Paspalum distichum* [33, 34]. Depending on population survival, *S*. *furcifera* naturally occurs in two phenotypically distinct forms, i.e., short- and long-winged. The short-winged adults lay more eggs are generated when the host plant abundance is sufficiently rich, whereas the long-winged adults migrate to find rice plants with higher nutritional quality. These behaviors of *S*. *furcifera* suggest a crucial role for chemosensation. However, the precise physiological mechanisms that mediate these important behaviors remain unknown at present.

Three decades ago, Obata et al. [35, 36] found that volatiles and extracts from rice appeared to attract three related Delphacidae rice planthopper species (*S*. *furcifera*, *Nilaparvata lugens*, and *Laodelphax striatellus*), but not when they lacked antennae in dark conditions. However, except for our previous reports on OBPs functional research [37, 38, 39], the molecular mechanisms of olfaction in Delphacidae are largely unknown. ORs bind odorants more specifically than OBPs, and a single OR is sufficient to change insect behavior, whereas a specific OBP is not needed to invoke behavioral change [37]. Thus, in the present study, we sequenced and analyzed the head transcriptome of *S*. *furcifera* adults using next generation sequencing, where we identified 63 OR and 14 IR transcripts in this pest insect species. We also conducted transcriptome sequencing and gene ontology (GO) annotation, as well as scanned sequences for motif-patterns and examined phylogenetic relationships.

## Results

### Transcriptome sequencing and sequence assembly

**Fig 1:**
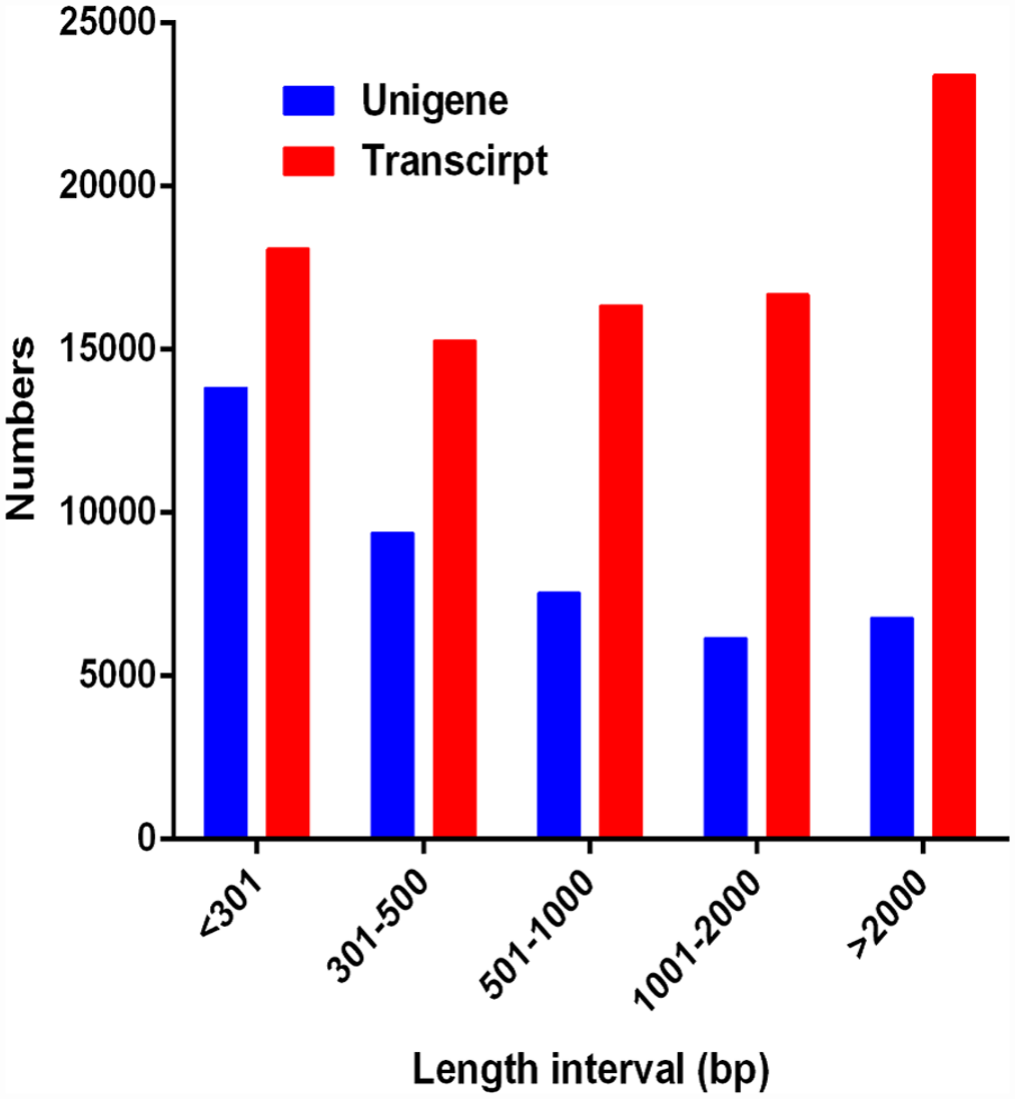
Distribution of transcripts and unigene length in the *S*. *furcifera* transcriptome assembly.

The *S*. *furcifera* head transcriptome was sequenced using the Illumina HiSeq^™^ 2000 platform and assembled with Trinity (v2012-10-05) (Table 1 and Fig 1). In total, about 163 million reads were obtained. After filtering, 142 million clean reads were generated, which comprised 14.2 gigabases (Gb), with a longest length of 28,290 nt and a median length of 456 nt. These reads were assembled into 89,810 transcripts and 43,712 unigenes, with N50 lengths of 3,014 and 2,217 nt, respectively (Table 1). In addition, the unigenes with a sequence length >1000 nt accounted for 29.63% of the transcriptome assembly (Fig 1). The transcriptome raw reads have been deposited with the NCBI SRA database (accession number: SRR2068690).

**Table 1:** Summary of *S*. *furcifera* transcriptome assembly.

Total size	14.2 Gb
Number of transcripts	89810
Total unigene count	43712
Genes with homologs in NR	14430
Total transcript nucleotides	139043608
Total unigene nucleotides	46173104
N50 transcript length	3014 nt
N50 unigene length	2217 nt
Longest unigene length	28920 nt
Median unigene length	456 nt
GC content	42.85%

### Homology analysis and GO annotation

**Fig 2:**
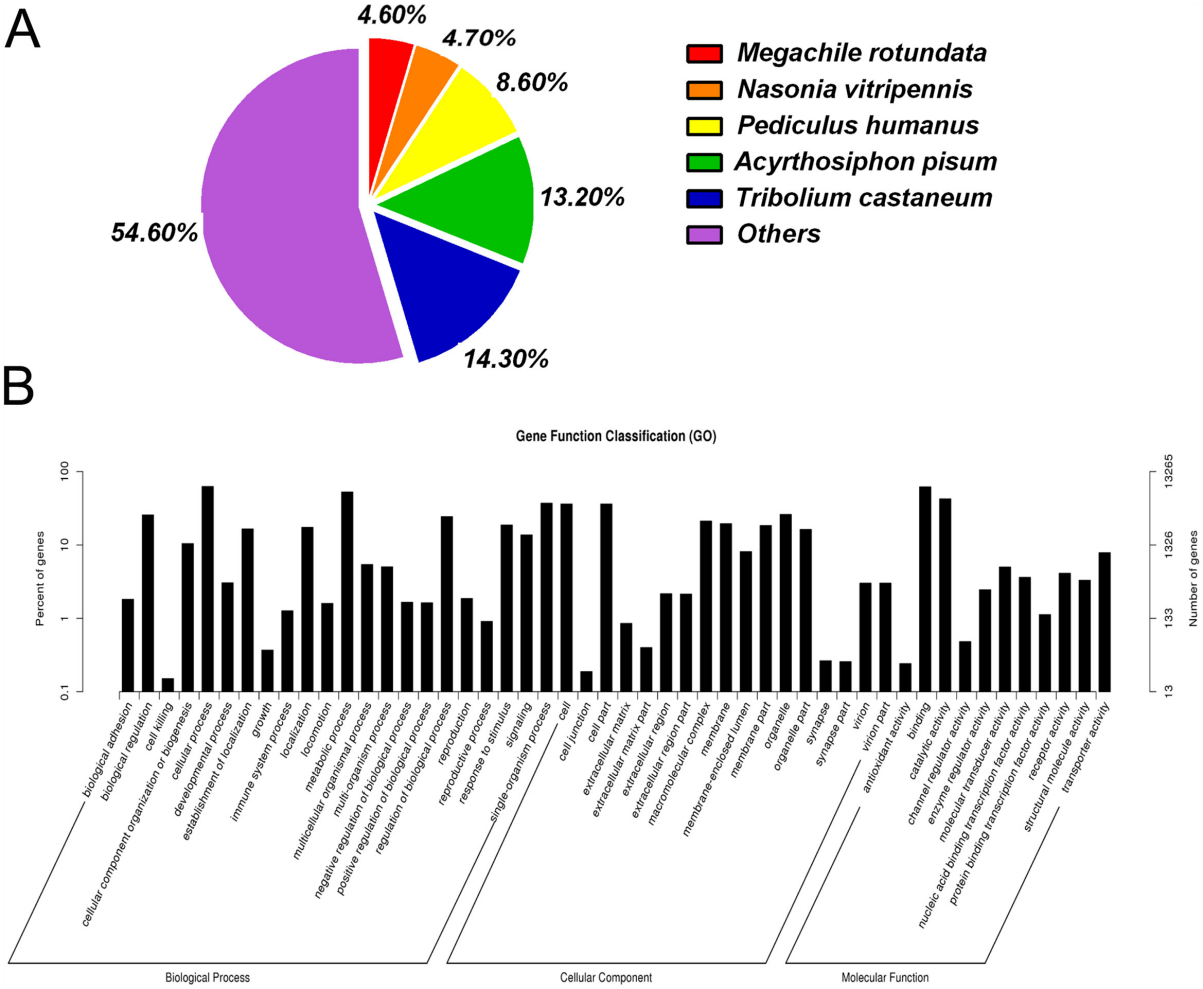
Annotation summaries for *S*. *furcifera* head unigenes. (A) Species distribution of unigenes with the best hit annotation terms in the NR database. (B) Gene ontology (GO) classifications of *S*. *furcifera* unigenes.

BLASTx homology searches of all 43,712 unigenes showed that 14,430 (33.1%) had homologous genes in the non-redundant (NR) protein database with a cut-off E-value of 10^−5^. The best match percentage (14.3%) was with *Tribolium castaneum* sequences, followed by sequences from *Acyrthosiphon pisum* (13.2%), *Pediculus humanus* (8.6%), *Nasonia vitripennis* (4.7%), and *Megachile rotundata* (4.6%) (Fig 2A).

GO annotations for all the unigenes were obtained using the Blast2GO pipeline according to the BLASTx search against NR. The GO annotations were used to classify the transcripts into functional groups according to specific GO categories. Among the 43,712 unigenes, 13,265 (30.3%) could be assigned to various GO terms. In the molecular function category, the genes expressed in the head were mostly enriched for binding (e.g., nucleotide, ion, and odorant binding) and catalytic activity (e.g., hydrolase and oxidoreductase). In the biological process category, the most common were cellular and metabolic processes. In the cellular component terms, the most abundant were cell and organelle (Fig 2B).

### Identification OR/IR genes

The unigenes related to candidate olfactory receptors (ORs/IRs) were identified based on keyword searches of the BLASTx annotations. The predicted protein unigene protein sequences were also analyzed using PSI-BLASTp with known aphid olfactory receptors [32, 38]. In total, we identified 77 unigenes that belonged to the olfactory receptor family in the head transcriptome of *S*. *furcifera*, including 63 ORs and 14 IRs, all of which shared similarity with other insect OR and IR genes. Among these, 27 OR and 3 IR genes encoded putative, complete opening reading frames. Further information for the OR and IR genes including the unigene references, lengths, and best BLASTx his are listed in Tables 2 and 3. To validate the reliability of the transcriptome assembly, we randomly chose 32 full-length ORs for RT-PCR validation. To cover a sequence that was as long as possible, the primers were designed to span the ORF, the primer sequences are listed in S1 Table. As a result, all 32 ORs were successfully amplified by RT-PCR (S1 Fig). The PCR results were confirmed by sequencing. All of the OR and IR sequences in this study are listed in S1 File.

**Table 2:** Unigenes of candidate odorant receptors.

Gene name	Gene id	Gene Length (bp)	Amino acid length	Full-length	NR ID	NR E value	NR Description
**Orco**	comp60837_c0	3496	473	yes	KC526964.1	5.29E-08	*Tribolium castaneum* or16 gene for olfactory receptor 16
**OR1**	comp61288_c0	1571	416	yes	ABQ84982.1	5.12E-09	putative chemosensory receptor 12 [*Spodoptera littoralis*]
**OR2**	comp61986_c0	1785	432	yes	XP_002067278.1	7.75E-09	PREDICTED: putative odorant receptor 94b-like [*Megachile rotundata*]
**OR3**	comp58780_c0	1772	427	yes	EEZ99241.1	3.50E-11	odorant receptor 28 [*Tribolium castaneum*]
**OR4**	comp57076_c0	1537	415	yes	EFN89949.1	2.86E-06	Putative odorant receptor 85d [*Harpegnathos saltator*]
**OR5**	comp63016_c1	1445	407	yes	EFA09246.1	4.16E-07	odorant receptor 15 [*Tribolium castaneum*]
**OR6**	comp61037_c0	1459	402	yes	ACX53766.1	4.45E-06	chemosensory receptor [*Heliothis virescens*]
**OR7**	comp58541_c0	1676	473	yes	XP_556129.1	5.22E-07	PREDICTED: putative odorant receptor 9a-like [*Apis florea*]
**OR8**	comp32401_c0	1490	399	yes	XP_001354859.2	2.81E-08	GL14885 [*Drosophila persimilis*] >gi|194107678|gb|EDW29721.1| GL14885 [*Drosophila persimilis*]
**OR9**	comp63582_c0	1640	402	yes	XP_002067278.1	4.00E-09	GI14807 [*Drosophila mojavensis*] >gi|193908681|gb|EDW07548.1| GI14807 [*Drosophila mojavensis*]
**OR10**	comp60973_c0	1673	412	yes	ABQ84982.1	1.16E-06	putative chemosensory receptor 12 [*Spodoptera littoralis*]
**OR11**	comp52202_c0	1709	219	5′ exon lost	NP_001177607.1	6.96E-07	odorant receptor 267 [*Nasonia vitripennis*]
**OR12**	comp57173_c0	1347	425	yes	XP_966790.1	5.82E-08	PREDICTED: similar to Odorant receptor 85d, putative [*Tribolium castaneum*] >gi|226334912|emb|CAM84018.1| olfactory receptor 20 [*Tribolium castaneum*]
**OR13**	comp59765_c0	1520	432	yes	NP_001177518.1	6.19E-06	odorant receptor 99 [*Nasonia vitripennis*]
**OR14**	comp59929_c0	1565	426	yes	NP_001166620.1	4.20E-12	odorant receptor 101 [*Nasonia vitripennis*]
**OR15**	comp50637_c1	798	225	5′exon lost	EAT45323.2	1.10E-10	olfactory receptor 9 [*Helicoverpa armigera*]
**OR16**	comp62723_c0	1674	466	yes	AFC91748.1	4.87E-10	putative odorant receptor OR40, partial [*Cydia pomonella*]
**OR17**	comp61628_c0	1478	434	yes	XP_003694963.1	4.87E-07	PREDICTED: putative odorant receptor 9a-like [*Apis florea*]
**OR18**	comp37650_c0	1561	398	yes	EFN71826.1	1.85E-14	Putative odorant receptor 24a [*Camponotus floridanus*]
**OR19**	comp59170_c0	1853	416	yes	NP_001164457.1	2.87E-06	odorant receptor 265 [*Nasonia vitripennis*]
**OR20**	comp64230_c0	2420	512	yes	EFZ21798.1	3.69E-11	Putative odorant receptor 13a [*Harpegnathos saltator*]
**OR21**	comp60687_c0	1451	277	5′exon lost	EHJ67075.1	3.14E-07	putative chemosensory receptor 10 [*Danaus plexippus*]
**OR22**	comp56083_c0	1281	323	yes	EAT37621.2	4.86E-11	Odorant receptor 9a, putative [*Aedes aegypti]*
**OR23**	comp147392_c0	1523	399	yes	EEZ99373.1	2.34E-06	Odorant receptor 2a [*Camponotus floridanus*]
**OR24**	comp60798_c0	2098	423	yes	EFN67925.1	8.65E-09	Putative odorant receptor 9a [*Camponotus floridanus*]
**OR25**	comp55722_c0	1603	396	yes	XP_002067278.1	7.97E-09	olfactory receptor, putative [*Aedes aegypti*] >gi|108873398|gb|EAT37623.1| AAEL010426-PA [*Aedes aegypti*]
**OR26**	comp57440_c0	1843	438	yes	EEZ99406.1	8.15E-12	odorant receptor 32 [*Tribolium castaneum*]
**OR27**	comp57217_c0	1952	493	yes	XP_003246096.1	3.79E-30	odorant receptor 57 [*Tribolium castaneum*]
**OR28**	comp49303_c0	765	193	5′exon lost	EFA09294.1	1.95E-08	odorant receptor 10 [*Tribolium castaneum*]
**OR29**	comp44516_c0	1543	412	yes	ACC63238.1	2.85E-08	GK16337 [Drosophila willistoni] >gi|194163363|gb|EDW78264.1| GK16337 [*Drosophila willistoni*]
**OR30**	comp48809_c0	592	164	5′exon lost	XP_002067278.1	1.65E-14	PREDICTED: odorant receptor 46a, isoform A-like [*Apis mellifera*]
**OR31**	comp56791_c0	1503	385	yes	EFN67929.1	4.68E-13	Odorant receptor 49b [*Camponotus floridanus*]
**OR32**	comp43248_c0	1409	434	3′exon lost	AFL03413.1	3.47E-09	odorant receptor 2 [*Locusta migratoria*]
**OR33**	comp51860_c0	728	221	5′exon lost	EFA09294.1	7.12E-16	odorant receptor 10 [*Tribolium castaneum*]
**OR34**	comp42791_c0	400	111	5′exon lost	XP_002015587.1	2.16E-09	Or43a [*Drosophila pseudoobscura pseudoobscura*] >gi|198135482|gb|EAL24773.2| Or43a [*Drosophila pseudoobscura pseudoobscura*]
**OR35**	comp52124_c0	1463	408	yes	XP_003704145.1	1.15E-18	PREDICTED: odorant receptor Or2-like [*Megachile rotundata*]
**OR36**	comp58617_c0	1753	429	yes	EEZ99404.1	4.86E-09	odorant receptor 29 [*Tribolium castaneum]*
**OR37**	comp39740_c0	588	185	5′exon lost	ACX53766.1	7.12E-06	chemosensory receptor [*Heliothis virescens*]
**OR38**	comp50926_c0	766	191	5′exon lost	EFA02940.1	7.88E-14	odorant receptor 47 [*Tribolium castaneum*]
**OR39**	comp57366_c0	1396	411	yes	XP_001850048.1	1.94E-06	Odorant receptor 9a [*Culex quinquefasciatus*] >gi|167867973|gb|EDS31356.1| Odorant receptor 9a [*Culex quinquefasciatus*]
**OR40**	comp572797_c0	203	68	5′3′exons lost	XP_003699516.1	4.03E-08	odorant receptor 9 [*Tribolium castaneum*]
**OR41**	comp51830_c0	847	273	5′exon lost	XP_003402693.1	3.83E-09	PREDICTED: odorant receptor 2a-like [*Bombus terrestris*]
**OR42**	comp53845_c0	1238	398	yes	NP_001177520.1	8.20E-12	odorant receptor 101 [*Nasonia vitripennis*]
**OR43**	comp47056_c0	436	145	5′exon lost	ACX53766.1	5.24E-09	chemosensory receptor [*Heliothis virescens*]
**OR44**	comp662599_c0	271	78	5′exon lost	NP_001164458.1	3.79E-06	odorant receptor 98 [*Nasonia vitripennis*]
**OR45**	comp49469_c0	1298	398	yes	EHJ70340.1	3.09E-14	putative chemosensory receptor 10 [*Danaus plexippus*]
**OR46**	comp49409_c0	781	234	5′exon lost	XP_002056218.1	1.32E-09	Odorant receptor 9a, putative [*Aedes aegypti*]
**OR47**	comp59794_c0	2202	431	yes	EEZ99412.1	2.05E-15	odorant receptor 44 [*Tribolium castaneum*]
**OR48**	comp53841_c0	1605	418	yes	XP_001651754.1	1.58E-09	odorant receptor [*Aedes aegypti*] >gi|108878225|gb|EAT42450.1| AAEL006005-PA [*Aedes aegypti*] >gi|197322752|gb|ACH69140.1| odorant receptor 9 [*Aedes aegypti*]
**OR49**	comp47025_c1	753	166	5′exon lost	EEZ99415.1	1.02E-09	odorant receptor 60 [*Tribolium castaneum*]
**OR50**	comp50910_c0	1518	425	yes	ADK48356.1	1.19E-06	odorant receptor 43a [*Drosophila melanogaster*] >gi|301032209|gb|ADK48416.1| odorant receptor 43a [*Drosophila melanogaster*]
**OR51**	comp43499_c0	1525	425	yes	CAD31949.1	1.70E-06	putative chemosensory receptor 8 [*Heliothis virescens*]
**OR52**	comp53588_c0	1613	464	yes	CAG38118.1	4.06E-23	putative chemosensory receptor 17 [*Heliothis virescens*]
**OR53**	comp33086_c0	667	196	5′exon lost	NP_001177509.1	3.62E-13	odorant receptor 69 [*Nasonia vitripennis*]
**OR54**	comp48912_c0	1539	462	yes	XP_003246096.1	1.42E-29	olfactory receptor, putative [*Aedes aegypti*]
**OR55**	comp47360_c0	1427	418	yes	NP_001177515.1	2.40E-16	odorant receptor 89 [*Nasonia vitripennis*]
**OR56**	comp36388_c0	570	166	5′exon lost	EEZ99409.1	1.18E-11	odorant receptor 36 [*Tribolium castaneum*]
**OR57**	comp26089_c0	689	229	5′3′exons lost	EEZ99404.1	2.23E-08	odorant receptor 29 [*Tribolium castaneum*]
**OR58**	comp524680_c0	323	72	5′3′exons lost	DAA05996.1	3.43E-06	TPA: odorant receptor 40 [*Bombyx mori*]
**OR59**	comp971_c0	1022	340	5′3′exons lost	NP_001091787.1	6.97E-11	olfactory receptor 10, partial [*Helicoverpa armigera*]
**OR60**	comp31797_c0	328	94	5′exon lost	NP_001116810.1	3.03E-10	olfactory receptor 46 [*Bombyx mori*]
**OR61**	comp3354_c0	1128	359	3′exon lost	EEZ99241.1	1.93E-07	odorant receptor 28 [*Tribolium castaneum*]
**OR62**	comp50731_c0	988	329	5′3′exons lost	EFA01342.1	5.84E-07	odorant receptor 171 [*Tribolium castaneum*]

**Table 3:** Unigenes of candidate ionotropic receptors.

Gene name (clade)	Gene id	Gene Length (bp)	Amino acid length	Full-length	NR ID	NR Evalue	NR Description
**IR1 (IR41a)**	comp10038_c0	230	76	5′3′exons lost	ADR64681.1	6.04E-19	putative chemosensory ionotropic receptor IR41a [*Spodoptera littoralis*]
**IR2 (IR84a)**	comp22409_c0	1139	379	5′3′exons lost	NP_649720.2	3E-23	ionotropic receptor 84a [*Drosophila melanogaster*]
**IR3 (IR8a)**	comp64862_c0	3730	889	yes	AHA80144.1	0	ionotropic receptor 8a [*Schistocerca gregaria*]
**IR4 (IR93a. 1)**	comp36999_c0	327	109	5′3′exons lost	AFC91753.1	5E-10	putative ionotropic receptor IR93a, partial [*Cydia pomonella*]
**IR5 (IR68a. 1)**	comp40424_c1	319	97	5′ exon lost	AIG51921.1	2E-16	ionotropic receptor, partial [*Helicoverpa armigera*]
**IR6 (IR93a. 2)**	comp668513_c0	369	112	5′3′exons lost	AFC91753.1	7e-19	putative ionotropic receptor IR93a, partial [*Cydia pomonella*]
**IR7 (IR25a)**	comp64840_c0	3272	940	yes	AFC91757.1	0	putative ionotropic receptor IR25a [*Cydia pomonella*]
**IR8 (undentified)**	comp48754_c0	825	251	5′ exon lost	AFC91765.1	2E-28	putative ionotropic receptor IR76b [*Cydia pomonella*]
**IR9 (IR40a. 1)**	comp54773_c0	2212	396	yes	NP_610140.4	7E-79	ionotropic receptor 40a, isoform F [*Drosophila melanogaster*]
**IR10 (IR68a. 2)**	comp571726_c0	270	90	5′3′exons lost	ADR64682.1	2E-32	putative chemosensory ionotropic receptor IR68a [*Spodoptera littoralis*]
**IR11 (IR68a. 3)**	comp904987_c0	354	117	5′3′exons lost	ADR64682.1	6E-15	putative chemosensory ionotropic receptor IR68a [*Spodoptera littoralis*]
**IR12 (IR76b)**	comp34339_c0	604	201	5′3′exons lost	NP_649176.1	5E-68	ionotropic receptor 76b [*Drosophila melanogaster*]
**IR13 (IR93a. 2)**	comp349927_c0	435	144	5′3′exons lost	AGJ51190.1	3E-21	olfactory ionotropic receptor IR93a *[Panulirus argus]*
**IR14 (IR40a. 2)**	comp556111_c0	319	105	5′3′exons lost	ADR64680.1	3.56E-32	putative chemosensory ionotropic receptor IR40a [*Spodoptera littoralis*]
**iGluR1**	comp61439_c0	5284	915	yes	XP_003708435.1	0	PREDICTED: glutamate receptor, ionotropic kainate 2-like [*Nasonia vitripennis*]
**iGluR2**	comp59047_c0	6217	965	yes	XP_003708429.1	0	PREDICTED: similar to CG11155 CG11155-PA [*Tribolium castaneum*]
**iGluR3**	comp54356_c0	3462	901	yes	XP_311343.4	0	predicted protein [Pediculus humanus corporis] >gi|212510560|gb|EEB13717.1| predicted protein [*Pediculus humanus corporis*]
**iGluR4**	comp804094_c0	363	103	5′3′exons lost	XP_003694299.1	5.44E-07	>gi|108872862|gb|EAT37087.1| AAEL010880-PA [*Aedes aegypti*]
**iGluR5**	comp1304517_c0	203	59	5′3′exons lost	BAD92087.1	1.28E-21	PREDICTED: glutamate [NMDA] receptor subunit epsilon-2-like, partial [*Ornithorhynchus anatinus*]

### Motif-pattern and phylogenetic trees analysis

**Fig 3:**
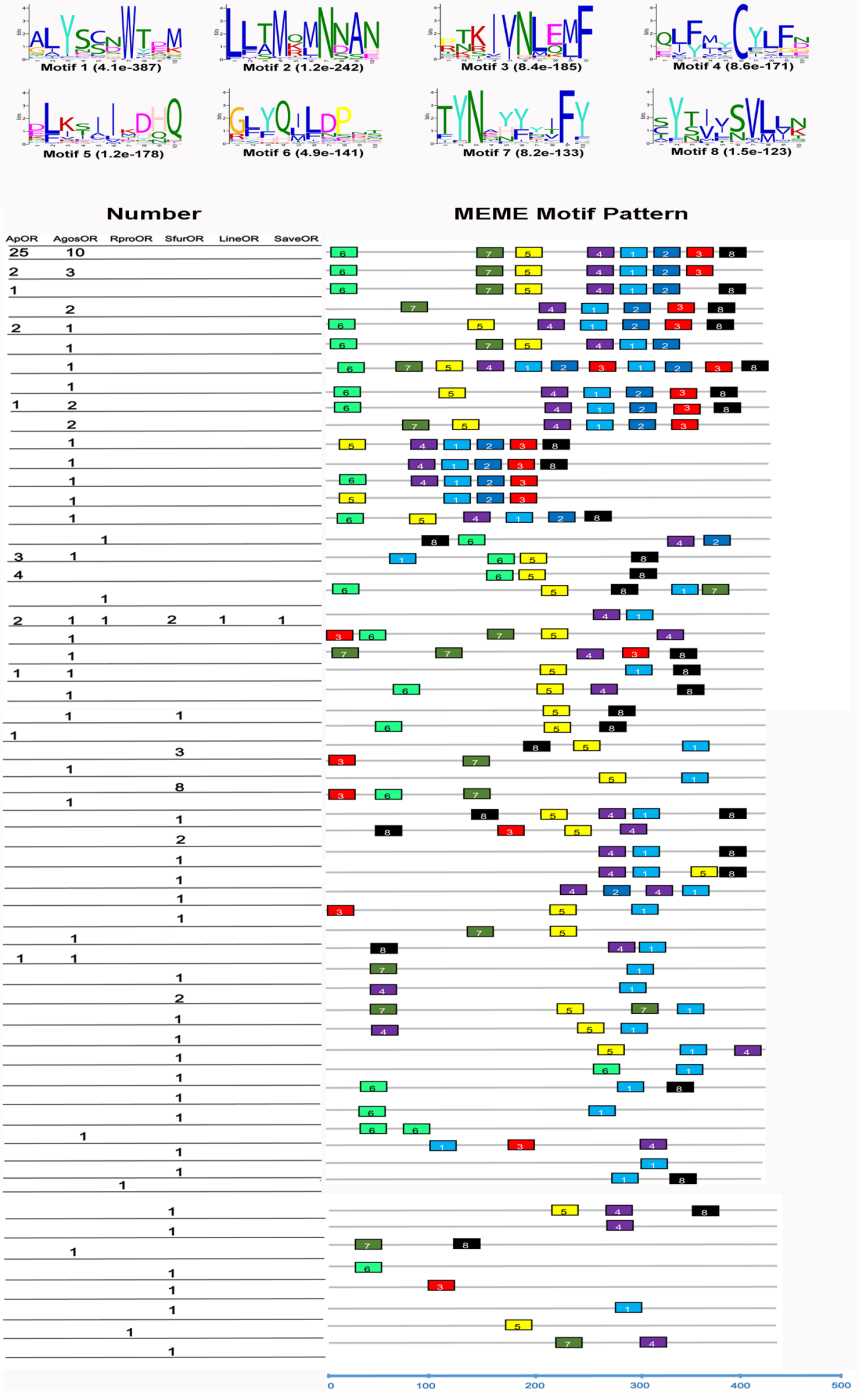
Motif analysis of ORs in the Hemipera. Parameters used for motif discovery were: minimum width = 6, maximum width = 10, maximum number of motif to find = 8. The upper parts listed the eight motifs discovered in the 130 ORs using MEME (version 4.9.1) on line server (http://meme.nbcr.net/meme/). The lower parts of different colors indicate approximate locations of each motif on the predicted protein sequence. The numbers in the boxes correspond to the numbered motifs in the upper part of the figure, where small number indicates high conservation. The numbers on the bottom showed the approximate locations of each motif on the protein sequence, starting from the N-terminal.

Conserved motifs are important elements of functional domains. We used the MEME server to identify conserved motifs in 130 hemiptera ORs. Parameters used in this and all other motif predictions of this study were: minimum width = 6, maximum = 10, maximum number of motif to find = 8. As a result, eight motifs (Most case occur: Motif-1, ALYSCNWTDM; Motif-2, LLTMQMNNAN; Motif-3, PTKIVNLEMF; Motif-4, QLFMYCYIFD, Motif-5, DLKSIIKDHQ; Motif-6, GHYQIIDPET, Motif-7, TYNAYYIFY; Motif-8, CYTVVSVLLN) were found for hemipteran ORs (Fig 3). Most motif amino acid residues locate in intramembrane domain, not in transmembrane domain. Motif 1, 4, 5 were the top three motifs present in these ORs, the ratio were 44.6%, 32.3% and 33.1%, respectively. We also carried out a motif-pattern analysis of hemipteran ORs. It was quite different between species with the exception of the ORco sequences, which exhibited the same “4–1” motif-pattern. The “6-7-5-4-1-2-3-8” pattern was the most common motif in aphids with 25 ORs in *A*. *pisum* and 10 ORs in *A*. *gossypii* exhibiting the pattern. The most prevalent motif pattern in *S*. *furcifera* was the “5–1” motif, which was found in 8 SfurORs.

**Fig 4:**
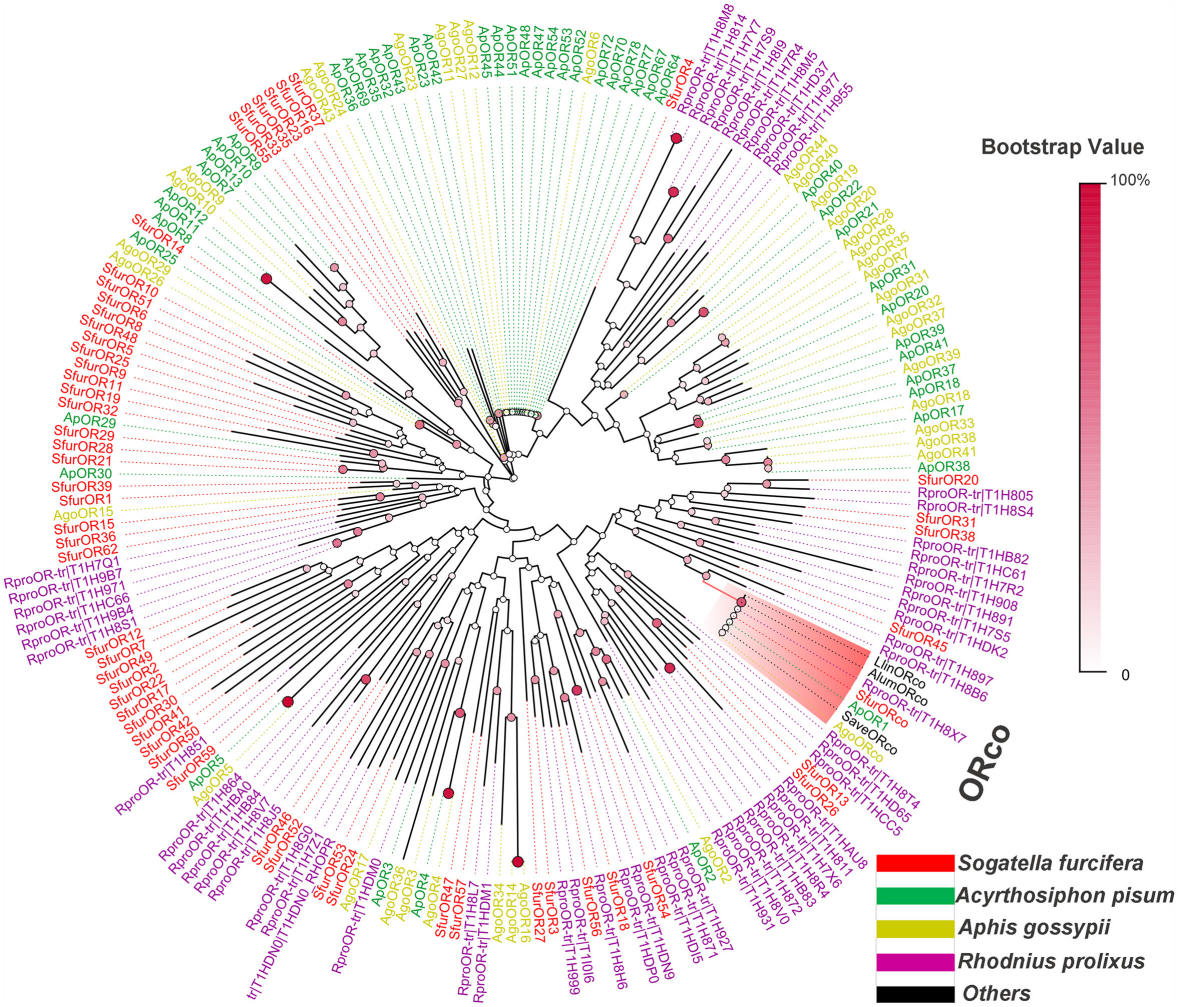
Phylogenetic tree of *S*. *furcifera* SfurORs and other hemipteran ORs. Species abbreviations: Ap, *A*. *pisum*; Ago, *A*. *gossypii*; Rpro, *R*. *prolixus*; Sfur, *S*. *furcifera*; Llin, *Lygus lineolaris*; Alum, *Apolygus lucorum*; Save, *Sitobion avenae*.

**Fig 5:**
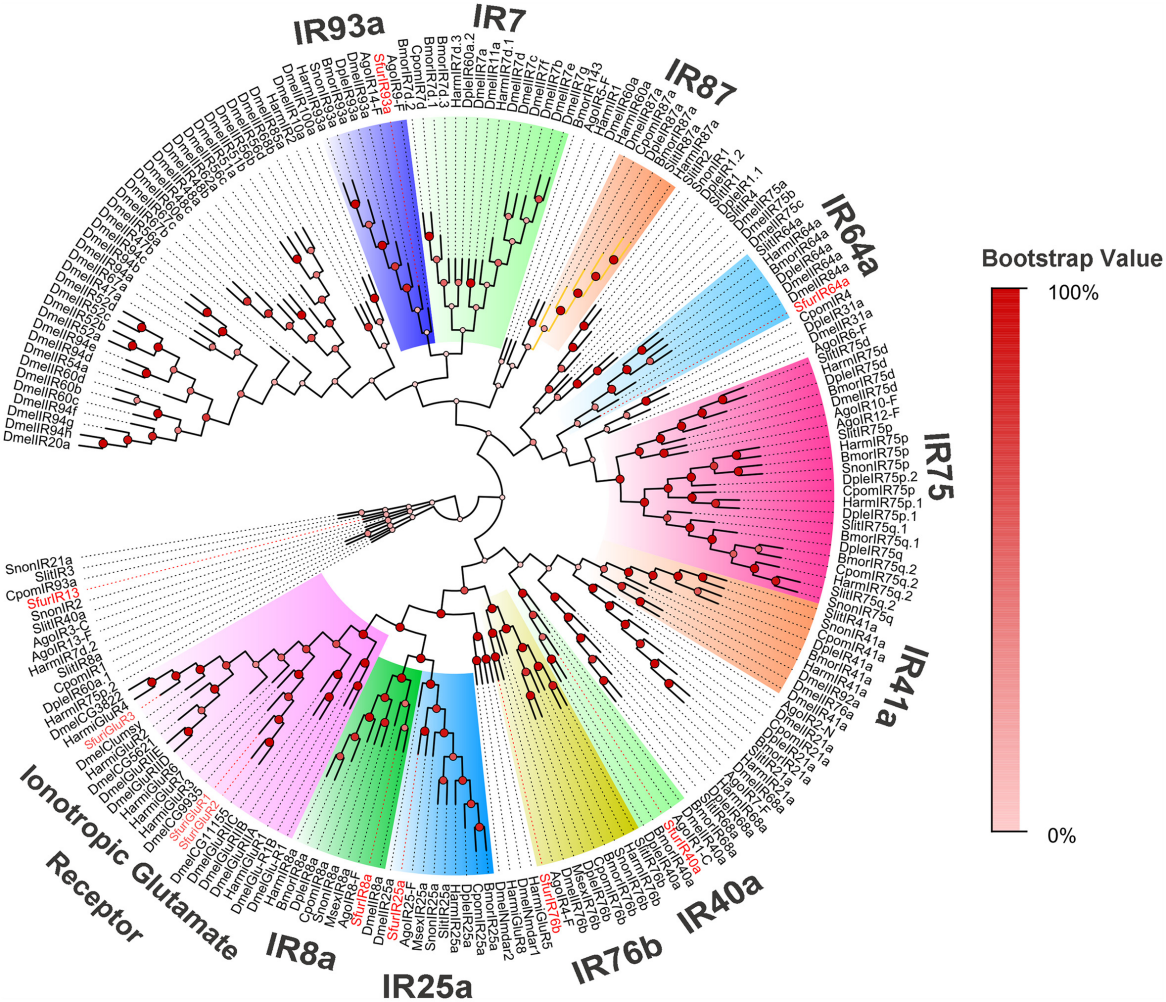
Phylogenetic tree of putative *S*. *furcifera* IRs, *Drosophila melanogaster* iGluRs and IRs, and other insect IRs. SfurIRs are highlighted in red letters. Bmor, *Bombyx mori*; Cpom, *Cydia pomonella*; Dmel, *D*. *melanogaster*; Dple, *Danaus plexippus*; Harm, *Helicoverpa armigera*; Msex, *Manduca sexta*; Slit, *Spodoptera littoralis*; Snon, *Sesamia nonagrioides*; Ago, *A*. *gossypii*.

To distinguish putative OR or IR functions, we also constructed two phylogenetic trees using 57 ORs (>150 aa), 7 IRs, and 3 iGluRs (>120 aa) from *S*. *furcifera*, as well as known hemipteran ORs (Fig 4) and other insect IRs (Fig 5). In the OR tree, the co-receptor ortholog of *S*. *furcifera* SfurORco was easily assigned because it shared high similarity with the conserved hemipteran co-receptors. Other ORs were assigned to various clades, which indicated their distinct functions. Most of the other SfurORs had orthologous genes, such as SfurOR21/28/29/ApOR29, SfurOR59/ApOR5/AgoOR5, SfurOR3/RproOR-trT1H999, SfurOR18/56/RproOR-trT1H8H6, and SfurOR20/RproOR-trT1H805. In the IR tree, all 7 SfurIRs and 3 SfuriGluRs were assigned to known insect IR clades, i.e., SfurIR3 (IR64a clade), SfurIR4 (IR8a clade), SfuriGluR1/2/3 (ionotropic glutamate receptor clade), SfurIR9 (IR25a clade), SfurIR10 (unidentified clade), SfurIR11 (IR40a clade), SfurIR14 (IR76b clade), and SfurIR15 (IR93a clade).

### OR/IR transcript expression levels

**Fig 6:**
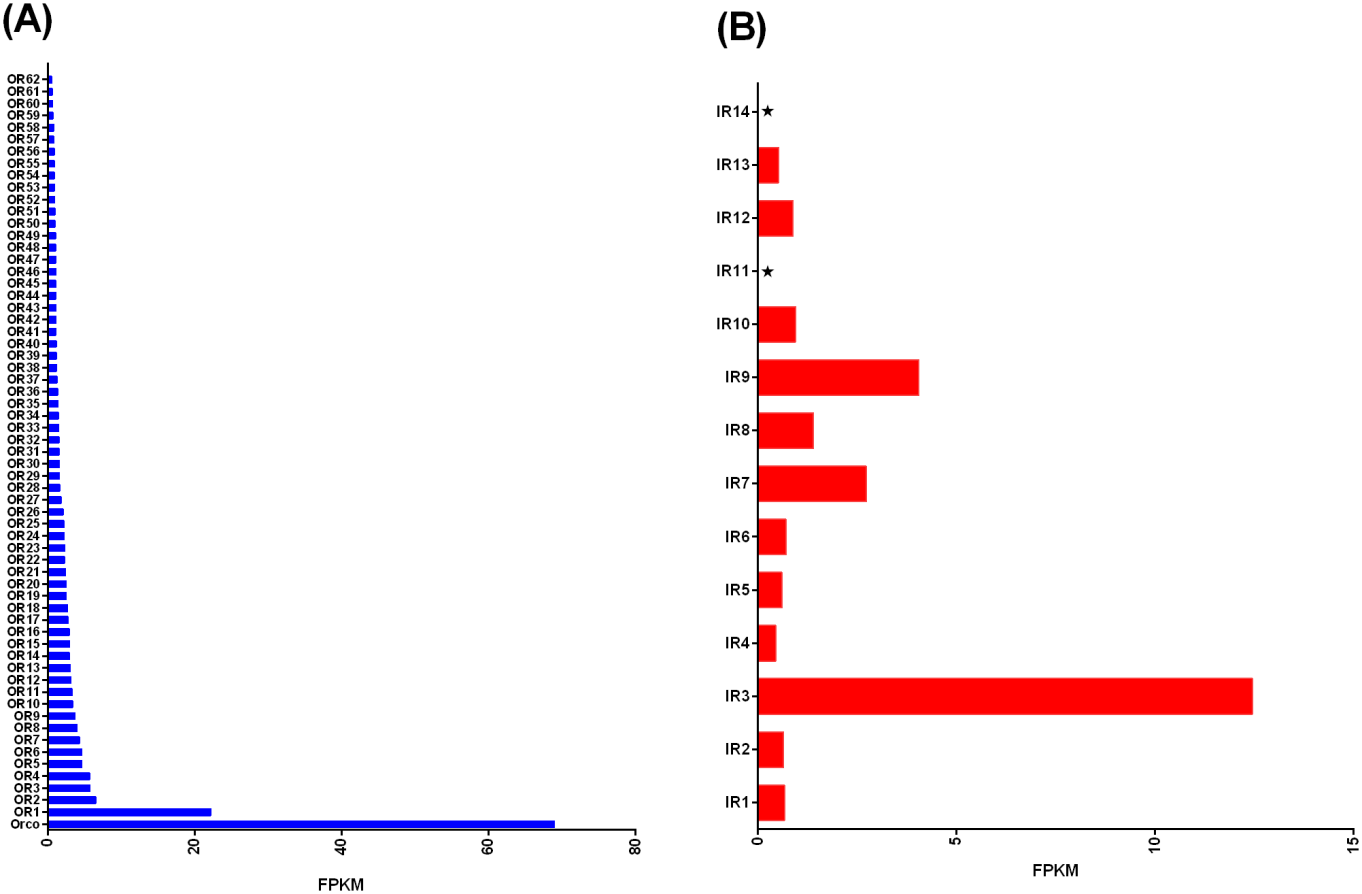
Comparison of ORs (A) and IRs (B) in the head according to Illumina read mapping. The asterisk indicates very low values. FPKM, expected number of fragments per kilobase of transcript sequence per million base pairs sequenced.

Among the 63 ORs, *SfurORco* had the highest expression level in the transcriptome data (fragments per kilobase per million mapped reads, FPKM = 68.96), followed by *SfurOR2* (FPKM = 22.21), *SfurOR3* (FPKM = 6.54), *SfurOR4* (FPKM = 5.73), and *SfurOR5* (FPKM = 5.72) (Fig 6A). For the 16 *SfurIRs*, *SfurIR3* had the highest expression level (FPKM = 12.46), followed by *SfurIR9* (FPKM = 4.06), and *SfurIR7* (FPKM = 2.73) (Fig 6B). *SfurIR11* and *SfurIR14* had very low expression levels in our transcriptome dataset.

## Discussion

In this study, we determined the repertoire of olfactory receptor superfamilies (ORs and IRs) in *S*. *furcifera* due to their potential significance as target genes for developing new pest control strategies, as well as for elucidating the molecular mechanisms that underlie insect-host plant interactions. In total, 14.2 Gb of *S*. *furcifera* head transcriptome data were sequenced, which is higher than that processed in most other studies [31, 32, 39–41]. After extensive sequencing and assembly using Trinity RNA-Seq, we identified 63 ORs and 14 IRs in *S*. *furcifera*. The number of ORs lies between that of two hemipteran aphids, *A*. *gossypii* (45 ORs) [32] and *A*. *pisum* (73 ORs) [38, 42], with sequenced genomes, and it is similar to the 62 ORs found in *D*. *melanogaster* [43] and the 79 ORs in *A*. *gambiae* [11, 12], but much lower than those in *T*. *castaneum* (259 ORs) [14] and *A*. *mellifera* (170 ORs) [13]. The number of IRs was similar to the 14 IRs found in *A*. *gossypii* [32], 18 in *D*. *melanogaster* [44], and 22 in *A*. *gambiae* [29], but slightly higher than those in *T*. *castaneum* (10 IRs) (these data were obtained from GenBank) and *A*. *mellifera* (nine IRs) (these data were obtained from GenBank). These findings suggest that the adaptation of distinct species to their plant hosts has led to the diversification of ORs and IRs during their evolution.

We conducted a MEME motif analysis using multiple hemiptera ORs to investigate differences among various species. Unlike insect OBPs [45], hemiptera ORs exhibit more differences, likely because ORs are more specific for odorant substrates than OBPs. In support of this, a single silkmoth pheromone receptor was activated by tis ligand to trigger sexual behaviors without the need of a specific OBP [37]. Furthermore, among various suborders of hemiptera the respective hosts are quite different, for example *R*. *prolixus* utilize blood meals and *S*. *furcifera* is an oligophagous pest that feeds only on few plants such as rice, maize. Thus we propose that they locate different hosts via volatiles based on their specific ORs. The exception, ORco, is more highly conserved than other ORs, which reflects its functional role in interacting with specific ORs to form the ligand—gated ion channel [17, 18]. mong the *SfurORs*, the *SfurORco* gene had the highest mRNA abundance, which is similar to *AgoORco* in *A*. *gossypii* [32]. In insects, the ORco gene is a co-receptor that forms a functional heteromer with specific ORs [17, 18]. In addition to *SfurORco*, the *SfurOR1* gene had higher expression levels than the other *SfurORs*, thereby suggesting that it may bind key plant host volatiles in *S*. *furcifera*, although further functional research is required to confirm this suggestion. The phylogenetic analysis of hemipteran ORs suggested that the SfurORs have undergone functional differentiation due to their scattered distribution. One specific *SfurOR* sub-clade, which included SfurOR16, 23, 33, 35, 37, and 55, had no counterparts in other species in this analysis, thereby suggesting that these six ORs may be activated by the specific host plant volatiles of *S*. *furcifera*.

To further distinguish putative IRs from iGluRs, the SfurIRs were aligned with IR orthologs from other insect species and some DmeliGluRs for BLASTx and phylogenetic analysis. We demonstrated that there were obvious differences in the distributions of DmeliGluRs and insect IRs. Like the ORco gene, the IR8a and IR25a genes are thought to act as co-receptors because of their co-expression with other IRs [46]. Our expression profiles were consistent with this hypothesis because IR3 (IR8), IR9 (IR40a.1), and IR7 (IR25a) were the top three genes among the14 *SfurIRs*, This result aslo agrees with the higher expression levels of *AgoIR8a* and *AgoIR25a* in *A*. *gossypii* [32].

In conclusion, based on analyses of head transcriptomic data, we identified 63 ORs and 14 IRs in the insect species *S*. *furcifera*. Our method was successful in identifying chemosensory receptor genes with low expression levels and our results provide a valuable resource for investigating and elucidating the mechanisms of olfaction in *S*. *furcifera*. As a crucial first step toward understanding their functions, we also conducted a comprehensive examination of the expression patterns of these olfactory receptor genes, which demonstrated that most of these OR and IR genes were expressed in chemosensory organs. Our findings provide the foundation for future research into the olfactory system of *S*. *furcifera* and for further investigations of classic behaviors, such as migration, as well as large numbers of potential target genes for controlling this pest.

## Materials and Methods

### Insect rearing and tissue collection

*S*. *furcifera* were collected from rice fields with the permission of the agricultural bureau in Libo county (25° 21’ N; 107° 49’ E), Guizhou province, China. The field studies did not involve endangered or protected species and no specific permissions were required for these insects. Collected insects were reared in the laboratory on rice seedlings at 26 ± 1°C, with a 16 h light: 8 h dark cycle. We collected 1000 heads of 1- to 3-day-old long-winged adults (male/female = 1/1) for transcriptome sequencing. We dissected various tissues (approximately 300 antennae, 150 mouthparts, 150 heads, 500 legs, and 50 bodies for each replicate) from long-winged adults under a microscope and we collected three replicates for each tissue type. The tissue samples were stored in RNAlater reagent (Qiagen, Valencia, CA, USA) at 4°C until further use.

### cDNA library construction and Illumina sequencing

Total RNA was extracted using TRIzol reagent (Invitrogen Carlsbad, CA, USA) according to the manufacturer’s protocol. The cDNA library construction and Illumina sequencing of the samples were performed by Novogene Bioinformatics Technology Co. Ltd, Beijing, China. The mRNA was purified from 10 μg of total RNA from *S*. *furcifera* heads using NEBNext oligo (dT)_25_ magnetic beads (NEB Next^®^ Poly(A) mRNA Magnetic Isolation Module, NEB, Beverly, MA, U.S.A.). NEBNext^®^ mRNA Library Prep Master Mix Set for Illumina^®^ (NEB, Beverly, MA, U.S.A.) was used for further library construction, mRNAs were fragmented into short sequences in the presence of RNA Fragmentation Reaction Buffer at 94°C for 5 min. Next, the first-strand cDNA was generated using Random Primer reverse transcription by using ProtoScript II Reverse Transcriptase (NEB, Beverly, MA, U.S.A.) at 25°C for 10 min, then 42°C for 15 min, and inactivation by heating at 70°C for 15 min, Second-strand cDNA using Second Strand Synthesis Enzyme Mix (NEB, Beverly, MA, U.S.A.) at 16°C for 2.5 hour with heated lid set at 40°C. Then NEBNext End Repair Enzyme Mix (NEB, Beverly, MA, U.S.A.) was used to perform end repair of the cDNA library at 30 minutes at 20°C. NEBNext dA-Tailing Reaction Buffer and Klenow Fragment (3’→5’ exo–) (NEB, Beverly, MA, U.S.A.) were used to dA-tail of cDNA Library at 37°C for 30 minutes. After end repair and dA-tailing, NEBNext Adaptor and USER^™^ enzyme, (NEB, Beverly, MA, U.S.A.) were used to ligate library DNA at 37°C for 15 minutes. After end repair and ligation of the adaptors, the products were amplified by PCR and purified using a QIAquick PCR Purification Kit to create a cDNA library, which was sequenced using the HisSeq^™^ 2000 platform.

### *De novo* assembly of short reads and gene annotation

After removing the adaptor sequences, low-quality reads, and reads where N ≥ 0.1%, the remaining reads were treated as clean reads. De novo transcriptome assembly was performed using the short reads assembly program Trinity (v2012-10-05) [47]. The overlap settings used for the assembly were 30 bp and 80% similarity, and all of the other parameters were set to their default values.

Unigenes >150 bp were aligned by BLASTx with protein databases, including Nr, Swiss-Prot, KEGG, and COG (e-value < 10^−5^), to identify protein with high sequence similarity and assign putative functional annotations. Next, we used the Blast2GO program [48] to obtain GO annotations of the unigenes and we obtained the GO functional classifications using WEGO software [49].

### Expression level analysis for the unigenes

The expression levels (abundances) of the unigenes were calculated with the FPKM method [50] using the formula: FPKM (A) = (10, 00, 000 × C × 1,000)/(N × L), where FPKM (A) is the expression level of gene A, C is the number of reads uniquely aligned to gene A, N is the total number of reads uniquely aligned to all genes, and L is the number of bases in gene A. The FPKM method can eliminate the influence of different gene lengths and sequencing discrepancies when calculating the abundance of expression.

### RNA extraction and cDNA synthesis

The approximately 300 *S*. *furcifera* headswere dissected and used for RNA extraction. The collected tissues were fast-frozen in liquid nitrogen and kept at -70°C for further use. Total RNA was extracted using a MiniBEST Universal RNA Extraction Kit (TaKaRa, Liaoning, Dalian, China) following the manufacturer’s instructions. The cDNA template was synthesized with Oligo(dT)18 primer as anchor primers, using PrimeScript^™^ I 1st Strand cDNA Synthesis Kit (TaKaRa, Liaoning, Dalian, China) at 42°C for 1 hr, The reaction was terminated by heating at 70°C for 15 min.

### PCR validation

Gene specific primers across ORF of selected OR genes were designed using “Primer Premier 5.0” for RT-PCR validation. The sequences of these primers are listed in Table A1. PCR experiments were carried out using a C-1000 thermacycler (Bio-Rad, Waltham, MA, USA), and Touchdown PCR reactions were performed under the following conditions: 94°C for 3 min; 20 cycles at 94°C for 50 sec, 60°C for 30 s, and 72°C for 2 min, with a decrease of the annealing temperature of 0.5°C per cycle. This was followed by 15 cycles at 94°C for 50 sec, 50°C for 30s, and 72°C for 2 min, and final incubation for 10 min at 72°C. The reactions were performed in 25 μl with 100 ng of single-stranded cDNA of *S*. *furcifera* heads, 2.0 mM MgCl_2_, 0.5 mM dNTP, 0.4μM for each primer and 1.25 U Taq polymerase or EX-Taq polymerase (TaKaRa, Liaoning, Dalian, China). PCR products were analyzed by electrophoresis on 1.5% w/v agarose gel in TAE buffer (40 mmol/L Tris—acetate, 2 mmol/L Na_2_EDTA·H_2_O) and the resulting bands visualized with ethidium bromide. DNA purification was performed using the TaKaRa MiniBEST Agarose Gel DNA Extraction Kit Ver.4.0 (TaKaRa, Liaoning, Dalian, China). Purified products were sub-cloned into a T/A plasmid using the pEASY-T3 vector system (TransGen Biotech, Beijing, China) following the manufacturer’s instructions. The plasmid DNAs was transformed into competent Trans1-T1 cells, positive clones were checked by PCR, and then sequenced by Sangon Biotech (Shanghai, China).

### Motif-pattern analysis

A total of 130 of hemipteran ORs were used for motif discovery and pattern analysis. The MEME (version 4.9.1) online server (http://meme.nbcr.net/meme/), which has been widely used for the discovery of DNA and protein motifs. The parameters used for motif discovery were as follows: minimum width = 6, maximum width = 10, and the maximum number of motifs to find = 8.

### OR/IR phylogenetic trees

MEGA 6.0 Beta [51] was used to construct two phylogenetic trees using 156 hemiptera ORs and 209 insect IR sequences (which is referred to IR phylogenetic tree in Liu, et al 2015 [52]) respectively with the neighbor-joining method. We also performed a bootstrap analysis of 1000 replicates to evaluate the branch strength in the phylogenetic tree. The OR dataset comprised ORs in from available databases: *A*. *gossypii* [32], *Acyrthosiphon pisum* [38, 42], and *Apolygus lucorum* ORco [53]. The IR dataset comprised IRs from: hemipteran species, i.e., *Aphis gossypii* [32], and *A*. *pisum* [38, 42]; lepidopteran species, i.e., *B*. *mori* [27], *C*. *pomonella* [54], *D*. *plexippus* [16], *M*. *sexta* [40], *S*. *littoralis* [55], and *S*. *nonagrioides* [41]; as well as IRs and iGluRs from the model insect *D*. *melanogaster* [27].

## Supporting Information

S1 Fig Agarose gel electrophoresis of RT-PCR verification. (DOCX)

S1 File SfurOR and SfurIR sequences. (FASTA)

S1 Table The RT-PCR primers. (DOC)
