# Supplementary figures and images for: Molecular Characterization and Differential Expression of an Olfactory Receptor Gene Family in the White-Backed Planthopper Sogatella furcifera Based on Transcriptome Analysis
Source: PLoS One. 2015 Nov 5;10(11):e0140605. doi: 10.1371/journal.pone.0140605 (PMC4634861; doi:10.1371/journal.pone.0140605)

**Supplementary file 2**


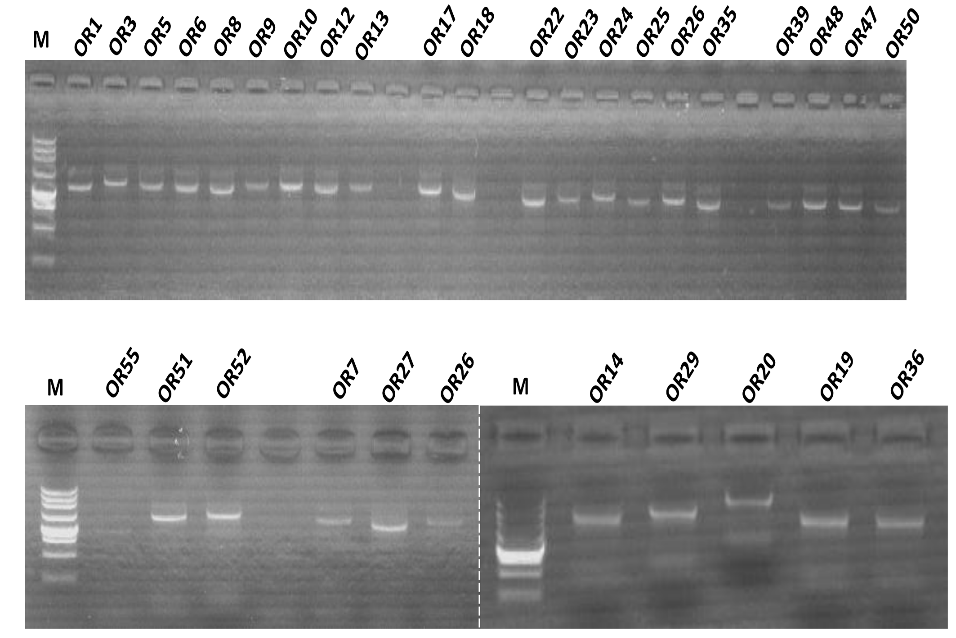


M: Marker, from bottom to top: 250, 500, 750, 1000, 1500, 2250, 3000, 4500

Supplement: S1 Fig — (DOCX) [file pone.0140605.s001.docx]
