# Supplementary material for: Molecular Characterization and Differential Expression of an Olfactory Receptor Gene Family in the White-Backed Planthopper Sogatella furcifera Based on Transcriptome Analysis
Source: PLoS One. 2015 Nov 5;10(11):e0140605. doi: 10.1371/journal.pone.0140605 (PMC4634861; doi:10.1371/journal.pone.0140605)
Supplement: S1 Table — (DOC) [file pone.0140605.s003.doc]

**Supplementary file 1**

| Gene  Name | 5’→3’ | Gene  Name | 5’→3’ |
| --- | --- | --- | --- |
| SfurOR1-F | acagcatgatgaagggtgactc | SfurOR1-R | atatcatgatccaggtcagagaatc |
| SfurOR3-F | acgttttgactttgacgttga | SfurOR3-R | gagaatattccctctagtgttgga |
| SfurOR5-F | tcttagcagatggacatgttctg | SfurOR5-R | aaactacagactaattattgaccaaac |
| SfurOR6-F | aattcataatggatgggaatgtg | SfurOR6-R | taatttttataccacttgggtcagtg |
| SfurOR7-F | gtaccttatgaatcttatattataggtagc | SfurOR7-R | ggaatgataaatagactcttatatcttctc |
| SfurOR8-F | tccatcgataagacagtgcc | SfurOR8-R | tgctgaaaatattacgagtgct |
| SfurOR9-F | ttgtggagtttggatgagtcc | SfurOR9-R | tcctgtttgagtgaatgatgaat |
| SfurOR10-F | ctcgatttattcaggaatatctcc | SfurOR10-R | gcaagctactttattgttcttcag |
| SfurOR12-F | atgcgacaaatattggtgcaaa | SfurOR12-R | tgattcaaattcgtttcttcaaagag |
| SfurOR13-F | ccgtgaacttgaacccgaaa | SfurOR13-R | gcctattgatgttattcatcgct |
| SfurOR14-F | cacagcataaactttcagagca | SfurOR14-R | ccggtatcagaatataggtacgta |
| SfurOR16-F | tgagattccttggaagtttgg | SfurOR16-R | tttttagcctgtagacctggtt |
| SfurOR17-F | ttgtgtaaaacacaatgatactgaa | SfurOR17-R | catcggagaacttgttgaaaat |
| SfurOR18-F | agtgaatgaaagtgaataaaagagg | SfurOR18-R | ttggtaattccatcaacagca |
| SfurOR19-F | tcccttccacggaacagttc | SfurOR19-R | gaatccagtcgcaaggttta |
| SfurOR20-F | aacaacaaaaacaacagaactaatcatgg | SfurOR20-R | gcttgtgcgttggatttatttaaactaa |
| SfurOR22-F | ttcaacccagcacatacctc | SfurOR22-R | gcaacaactttttattcacacaa |
| SfurOR23-F | gtactgctttcttatttaagtggatt | SfurOR23-R | gtactgctttcttatttaagtggatt |
| SfurOR24-F | ggacttccacaaataataatataggt | SfurOR24-R | agtagcactccagttccatcc |
| SfurOR25-F | tttgtaaacttagggtaggctgg | SfurOR25-R | tgatgactgttgatgttattgactg |
| SfurOR26-F | atcccaaataggtggaattaag | SfurOR26-R | gcaatgttttctatgatatcgc |
| SfurOR27-F | tttaagtaaacatccatttctccc | SfurOR27-R | ttatgacttatttagcattggca |
| SfurOR29-F | ctacttttcccgtacccatataa | SfurOR29-R | gatgcttgaactcacgtcca |
| SfurOR35-F | tcaaaccgtcagcagaccca | SfurOR35-R | tttcctcatgttgtacttatgtagc |
| SfurOR36-F | cattgggaaatcaggactttg | SfurOR36-R | catcatcagtctgatcgacaact |
| SfurOR39-F | tcagagattgtactgatctagtgatg | SfurOR39-R | gtaaacgatatactgattagaacgaa |
| SfurOR47-F | gcttgaatgggaataagtgttt | SfurOR47-R | caatagtgatgactggttgatgat |
| SfurOR48-F | tacacagtcgaataaaaaatccc | SfurOR48-R | gctaatgctaatagacctcaatga |
| SfurOR50-F | gaataataaaaatgagcatggca | SfurOR50-R | tgtgtgtaaggcaggtggat |
| SfurOR51-F | gactcctataatcatgaggcaag | SfurOR51-R | cataaaacaagaggtaaaccaaac |
| SfurOR52-F | tgttgagagtgctgattggaa | SfurOR52-R | actattgatgttgatacgctgatat |
| SfurOR55-F | actatgtacgacagtttgagacga | SfurOR55-R | cacgaacttgcttactcggt |

F: Forward, R: Reverse
